# Impact of COVID-19 Lockdown on Preterm Births, Low Birthweights and Stillbirths: A Retrospective Cohort Study

**DOI:** 10.3390/jcm10235649

**Published:** 2021-11-30

**Authors:** Charles Garabedian, Ninon Dupuis, Christophe Vayssière, Laurence Bussières, Yves Ville, Benoît Renaudin, Louise Dugave, Norbert Winer, Nathalie Banaszkiewicz, Patrick Rozenberg, Manon Defrance, Marie-Laure Legris, Thibaud Quibel, Philippe Deruelle

**Affiliations:** 1 CHU Lille, Department of Obstetrics, F 59000 Lille, France; louise.dugave.etu@univ-lille.fr; 2 University Lille, ULR 2694 METRICS, F 59000 Lille, France; 3 Department of Obstetrics and Gynecology, Paule de Viguier Hospital, CHU Toulouse, 31059 Toulouse, France; dupuis.ninon@gmail.com (N.D.); christophe.vayssiere@gmail.com (C.V.); 4 UMR1295 CERPOP (Centre for Epidemiology and Population Health Research), Team SPHERE (Study of Perinatal, Paedriatric and Adolescent Health: Epidemiological Research and Evaluation), Toulouse III University, 31062 Toulouse, France; 5 Obstetrics, Fetal Surgery, Medicine and Imaging Department, AP-HP, Hôpital Necker-Enfants Malades, 75007 Paris, France; laurence.bussieres@gmail.com (L.B.); yves.ville@aphp.fr (Y.V.); benoit.renaudin@aphp.fr (B.R.); 6 EHU 7328 PACT, Université de Paris, 75006 Paris, France; 7 Obstetrics and Gynecology Department, Centre Hospitalier Universitaire de Nantes, University Nantes, 44035 Nantes, France; norbert.winer@chu-nantes.fr (N.W.); Nathalie.BANASZKIEWICZ@chu-nantes.fr (N.B.); 8 UMR PhAN 1280 NUN INRAE, F-44000, University Nantes, 44035 Nantes, France; 9 Department of Obstetrics and Gynecology, Poissy-Saint Germain Hospital, 78300 Poissy, France; patrick.rozenberg@ght-yvelinesnord.fr (P.R.); manon.defrance.78@gmail.com (M.D.); Thibaud.Quibel@ght-yvelinesnord.fr (T.Q.); 10 UVSQ, Inserm, Team U1018, Clinical Epidemiology, CESP, Paris Saclay University, 78180 Montigny-le-Bretonneux, France; 11 Department of Obstetrics and Gynecology, University Hospital of Strasbourg, Avenue Moliere, 67000 Strasbourg, France; marie-laure.legris@chru-strasbourg.fr (M.-L.L.); pderuelle@unistra.fr (P.D.)

**Keywords:** pregnancy, preterm birth, stillbirth, low birthweight, COVID-19 pandemic

## Abstract

Objective: The effect of lockdowns during the coronavirus (COVID-19) pandemic on pregnancy outcomes remains uncertain. We aimed to evaluate the association between the COVID-19-related lockdown and pregnancy outcomes in maternity hospitals in France. Study design: This was a retrospective cohort study from six tertiary referral hospitals in different regions of France. Three 55-day periods were compared: pre-lockdown from 22 January 2020, lockdown from 17 March 2020, and post-lockdown from 11 May 2020 to 4 July 2020. We included all women who delivered singleton or multiple pregnancies, who delivered at ≥24 weeks of gestation and with birthweights ≥500 g. We documented gestational ages at the delivery of liveborn and stillborn infants (‘stillbirths’). These were categorized as having a very low birthweight (VLBW, <1500 g), or a low birthweight (LBW, <2500 g). Adjustments were made for place of birth, maternal age, parity and diabetes, and hypertensive disorders, as well as for multiple pregnancies. Results: In total, 11,929 women delivered in the six selected centers. This figure is constituted of 4093, 3829, and 4007 deliveries in the pre-lockdown (1), peri-lockdown (2), and post-lockdown (3) periods, respectively. There were no differences in pregnancy outcomes between these three periods. Overall, birth rates <27^+6^ weeks, between weeks 28^+0^ and 31^+6^, and between 32^+0^ and weeks 36^+6^ were 1.0%, 1.9%, and 4.4%, respectively. After adjustment, these rates were stable between periods 1 and 2 (adjusted odds ratio, aOR 0.90; 95% confidence interval, CI 0.69–1.19) and between periods 2 and 3 (aOR 1.04; 95% CI 0.80–1.36). Although more VLBW neonates were born during lockdown (3.5% vs. 2.6%, *p* = 0.03), this difference did not persist after adjustment (aOR 0.84, CI 95% 0.64–1.10). The LBW rates were similar during the three periods at 12.5% overall. The stillbirth rate was unaffected by the lockdown. Conclusion: The pregnancy outcomes (preterm birth, LBW, VLBW, and stillbirth rates) were not modified by the COVID-19 lockdown in our cohort study in France. Considering the discrepancies in results and methodological issues in previous published studies, there is not sufficient evidence to conclude that such lockdowns have any impact on perinatal outcomes.

## 1. Introduction

Lockdowns have been used by several health authorities to mitigate the effects of the COVID-19 pandemic on public health, which was caused by strains of the SARS-CoV-2 virus [1]. Several publications from Australia, the United States, Italy, Denmark, the Netherlands, Ireland, and England have reported substantial reductions in the preterm birth and/or low birthweight (LBW) rates following national COVID-19 lockdown measures [2,3,4,5,6]. According to these studies, the significant decrease in late preterm infants during the lockdown period could be attributed to lifestyle changes including resting at home, reduced physical activity, less shift work, less work-related stress, optimization of sleep durations, substantial reductions in air pollution, social distancing resulting in fewer infections by common pathogens, and an increased focus on hygiene. However, other publications from England and the USA did not support this observation and reported no difference in the overall rate of preterm birth at less than 37 weeks of gestation, nor any differences in the rate of delivery at less than 34, less than 32, or less than 28 weeks [7,8]. By contrast, one serious concern possibly associated with lockdown is the significant increase in the incidence of stillborn infants (‘stillbirths’) and neonatal mortality observed by several authors in England, Italy and Nepal [3,8,9]. This increase in the stillbirth rate does not seem to be a direct consequence of COVID-19 infection. It could reflect induced life changes caused by lockdown. In particular, it could be associated with a reduction in visits to hospitals or a reluctance to visit the hospital due to the fear of contracting COVID-19. This could result in delayed or deferred interventions in pregnancy. However, this increase in the incidence of stillbirths also remains controversial because other studies in England, Australia and Ireland reported no increased stillbirth rates associated with lockdown [2,5,10]. 

Thus, the potential effects of lockdowns on perinatal outcomes (and the magnitude of these effects), remain debatable. The objective of our study was to evaluate the adverse impacts of France’s lockdown on perinatal outcomes in a large population of pregnant women who were treated at six tertiary centers in different regions of France.

## 2. Materials and Methods

### 2.1. Setting

A nationwide lockdown was implemented in France on 17 March 2020, in response to the COVID-19 pandemic, and continued until 10 May 2020. The population was asked to stay at home or to limit mobility drastically, to follow strict hygiene measures with handwashing, and to practice social distancing. During the lockdown, offices, shops, colleges, schools, and all institutions considered as nonessential were closed. However, after the government announced the end of the lockdown, some nonessential services remained closed, and the population was still asked to restrict social connections and to limit their mobility. This was still the case until the end of our study. These measures were similar in every district of France.

### 2.2. Study Design

This was a retrospective cohort study comparing three equal periods: (1) pre-lockdown from 22 January to 16 March 2020, (2) lockdown from 16 March to 10 May 2020, and (3) post-lockdown from 11 May to 4 July 2020. All singleton or multiple pregnancies delivering at ≥24 weeks of gestation with a birthweight ≥500 g (as required in these maternity hospitals to manage pregnancies) during the study period in six tertiary referral centers across France were included. Cases of termination of pregnancy were excluded. The six centers were located in: Lille in the north, Nantes in the west, Toulouse in the southwest, Strasbourg in the east, Poissy in Greater Paris, and Necker in central Paris. The six centers accounted for 3.8% of all births in France in 2020 (28,026/740,000). During the lockdown, COVID-19 spread heterogeneously, and therefore, the pressures on local healthcare systems varied. Indeed, the east of France and the region of Paris faced a critical situation, with intensive care units overwhelmed with COVID-19 patients, whereas the north, west, and southwest were initially spared this pressure [11]. 

### 2.3. Outcomes

The following data were collected prospectively and analyzed retrospectively though computerized medical records: maternal age, parity (nulliparous versus multiparous women), singleton or multiple pregnancies, pre-pregnancy body mass index (BMI), diabetes with insulin required (gestational or preexisting diabetes), and hypertensive disorders.

Births were categorized according to gestational age in weeks and days (in superscript): extremely premature (23^+0^–27^+6^), very premature (28^+0^–31^+6^), moderate-to-late premature (32^+0^–36^+6^), term (37^+0^–41^+6^), and post-term (>42^+0^). Determination of the gestation period for all women included in the study was based on the results of the first trimester ultrasound scan, routinely performed in France, or from the date of oocyte retrieval in pregnancies generated using assisted reproductive technology (ART). Birthweight was categorized as very low (VLBW, <1500 g), or low (LBW, <2500 g). Elective delivery was defined as delivery after the induction of labor or during pre-labor Cesarean deliveries. Stillbirths at ≥24 weeks of pregnancy were also included in this study.

### 2.4. Statistical Analysis

Results were compared between different periods: Period 1 = pre-lockdown (from 22 January to 16 March 2020); Period 2 = lockdown (from 16 March to 10 May 2020); and Period 3 = post-lockdown (from 11 May to 4 July 2020). Categorical variables were presented as absolute numbers and percentages, and comparisons made using chi-squared or Fisher’s exact tests. Continuous variables were presented as medians with first and third interquartile ranges. Comparisons of continuous variables were made using Student’s *t* tests or Mann–Whitney nonparametric *U* tests. Adjusted odds ratios (aORs) and 95% confidence intervals (CIs) were estimated by a logistic regression of places of births, maternal age, parity, multiple pregnancies, diabetes, and hypertensive disorders. Moreover, as a delay in the management of pregnancies complicated with VLBW or LBW was possible, Kaplan–Meier survival curves were calculated for gestational ages at delivery within each period. All analyses were performed with RStudio version 1.0.136 (https://www.rstudio.com/products/rstudio/download/, accessed on 25 November 2021) assuming a significance level of α = 0.05.

## 3. Results

During the study period, 12,154 women delivered after 24 weeks of pregnancy in the six centers. Of these women, 174 were excluded, as the pregnancies were terminated for medical indications (1.4%). Fifty-one home births which led to admission in one of the six maternity hospitals were also excluded (*n* = 16, 16 and 19 during periods 1–3, respectively, with no significant differences; *p* > *0*.05). Finally, 11,929 women were included during the study period, with 4093, 3829, and 4007 women in periods 1–3, respectively. There were 2, 33, and 2 hospitalized patients with a record of laboratory-confirmed SARS-CoV-2 virus infection in the six centers during periods 1, 2, and 3, respectively.

Table 1 shows the baseline characteristics of the population. There were no significant differences regarding maternal age, parity, multiple pregnancies, BMI, or smoking status between study periods.

Pregnancy outcomes are summarized in Table 2 and Table 3. There were no differences in pregnancy outcomes between the three periods. The median gestational age at delivery was 39.3 weeks. Overall, birth rates at <27^+6^ weeks, between 28^+0^ and 31^+6^ weeks, and between 32^+0^ and 36^+6^ weeks of pregnancy were 1.0%, 1.9%, and 4.4%, respectively. After adjustment for place of birth, maternal age, parity, multiple pregnancies, diabetes, and hypertensive disorders, these rates were stable between periods 1 and 2 (aOR 0.90; 95% CI 0.69–1.19) or between periods 2 and 3 (aOR 1.04; 95% CI 0.80–1.36; Table 3). Table 4 shows the type of premature delivery (spontaneous vs. elective) during the study periods. The rate of elective delivery was similar during period 2 compared to period 1 (53.6% vs. 60.0%, *p* = 0.05), with this rate remaining stable during period 3 (60.0%).

**Figure 1:**
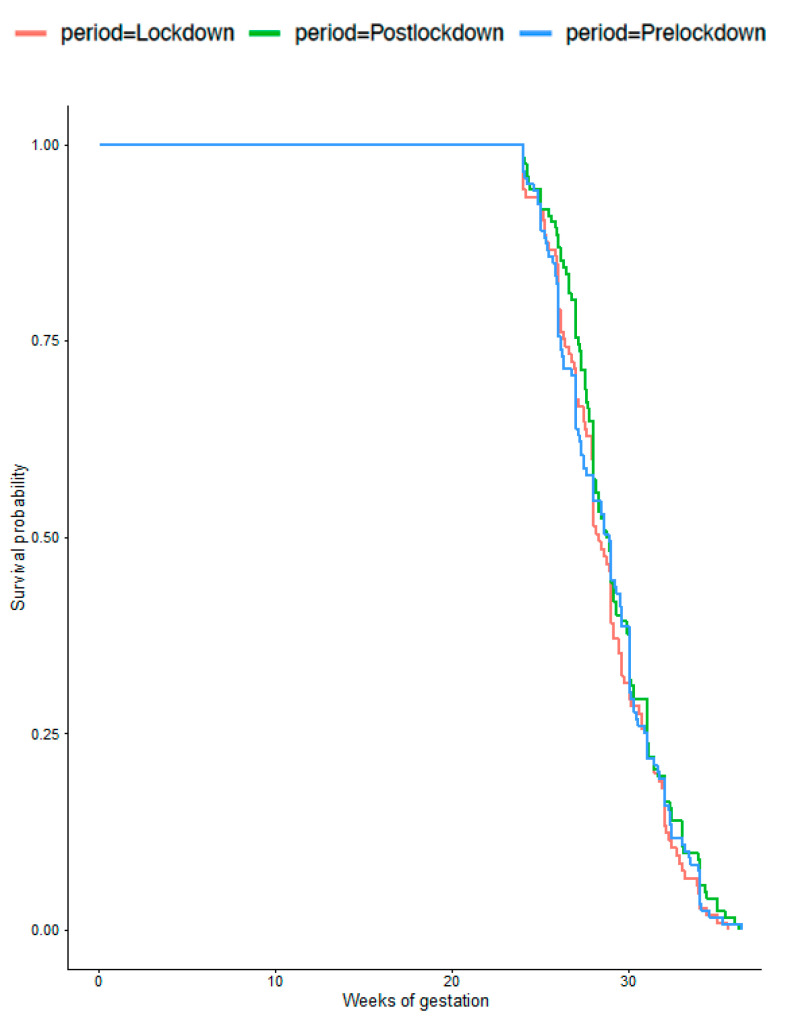
Gestational age at delivery for women with very low birthweight infants (<1500 g) (*p* = 0.7).

**Figure 2:**
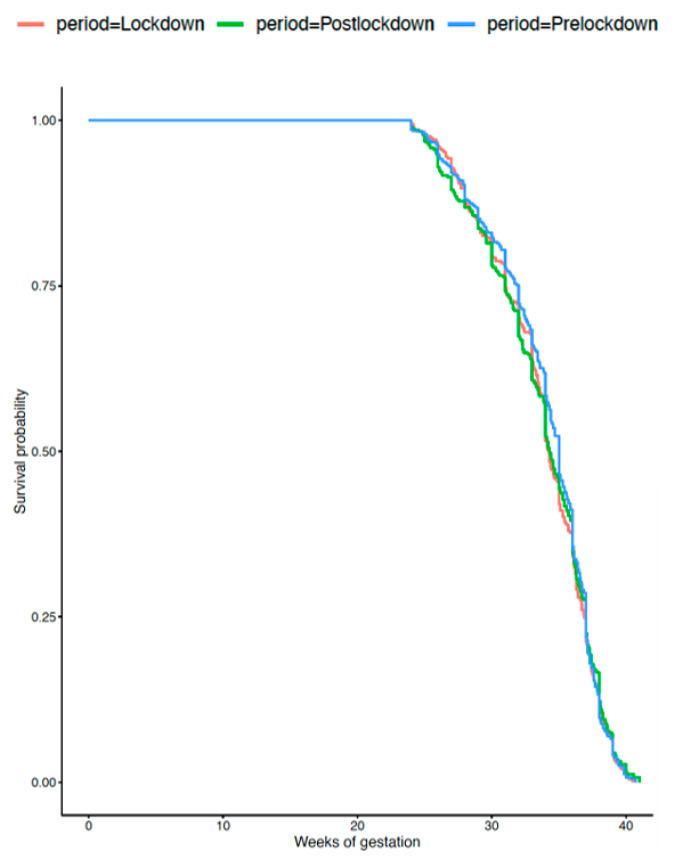
Gestational age at delivery for women with low birthweight (<2500 g) (*p* = 0.5).

The birthweight distributions were similar over the three periods (*p* = 0.07). Although more VLBW neonates were born during the lockdown compared with before (3.5% vs. 2.6%, *p* = 0.03), this difference did not persist after adjustment, as described above (aOR 0.84; 95% CI 0.64–1.10). The LBW rates were similar during the three periods at 12.5% overall. Figure 1 and Figure 2 present gestational ages at delivery for the VLBW and LBW infants and gestational age at delivery was also similar in these populations. The rate of stillbirth was unaffected by the lockdown, with a global rate of 0.5% and after adjustment with aOR 0.81; 95% CI 0.39–1.37 and aOR 1.05; 95% CI 0.52–2.12 between periods 1 and 2, and between 2 and 3, respectively (Table 3). 

## 4. Discussion

### 4.1. Main Findings

In our cohort study, preterm birth, low birthweight, and stillbirth rates remained unchanged before, during, and after the COVID-19 lockdown. These results were similar within the six maternity hospitals, even though they were affected differently from pressure on the healthcare system during the COVID-19 outbreak.

### 4.2. Interpretation

Since the beginning of the COVID-19 pandemic, several reports have showed decreases in the rates of preterm births, which appeared to be associated with the lockdown [2,3,4,5,6]. In a national quasi-experimental study in the Netherlands, Been et al. found reductions in the incidence of preterm birth across various time windows surrounding the implementation of COVID-19 lockdowns (e.g., an odds ratio of 0.77 95% CI 0.66–0.91, *p* = 0.0026) comparing 2 months after and 2 months before 9 March) [6]. In Denmark, Hederman et al. found a 90% decrease in the incidence of extremely preterm births (<28 weeks) during the lockdown period from 12 March to 14 April, compared with the same period during the previous 5 years [4]. In Ireland, Philip et al. focused on regional trends of VLBW and extremely LBW (ELBW) infants in one designated health area of Ireland over two decades, comparing periods of January–April from 1 January 2001 to 30 April 2020 [5]. They observed a 73% decrease in the incidence of very low birthweight infants, with no ELBW infants admitted to their regional neonatal intensive care unit. On the contrary, in a population-based prevalence proportion regional study in Spain, Arnaez et al., did not find any decrease in preterm rates during the lockdown period (15 March–21 June 2020) with respect to the whole pre-lockdown period (1 January 2015–14 March 2020) or to the pre-lockdown comparison periods (15 March–3 May 2015–2019): 6.5% (95%CI 5.6–7.4), 6.6% (95%CI 6.5–6.8), and 6.2% (95%CI 5.7–6.7), respectively. Such negative results were also observed in different settings: Wood et al. in four hospitals in Massachusetts [7] and Khalil et al. in a single center in London [8]. In our study, the difference observed in spontaneous versus elective delivery rates in preterm birth between the pre-lockdown period and the lockdown period is not supported with the same comparisons between the post-lockdown period and the lockdown period. Moreover, this difference was not found for preterm births before 32 weeks of gestation in either LBW or VLBW. Therefore, this significant difference might be an incidental finding.

Other studies with fewer numbers of cases suggested that lockdown was not associated with reduced rates of preterm birth or with low birthweight [7,8]. Studies assessing the increase of the incidence of stillbirths during the lockdown contradict one another [2,3,5,8,9]. Indeed, Matheson et al. have shown a decrease in Australia in three maternity hospitals in an interrupted time-series analysis between 1 January 2018 and 30 September 2020 [2]. Khalil et al. reported a 5.79-fold increase in stillbirth during lockdown in south London ([95% CI, 1.54–10.1]; *p* = 0.01), while Stowe et al. did not report any difference at the national or inter-regional level in the UK [8,10].

Several methodological issues might help to explain these discrepancies. Indeed, evaluating changes in gestational age over time and comparing preterm birth rates is challenging. In particular, according to the period selected and the length of time involved in these previous studies, differences in maternal demographics, clinical characteristics and other unmeasured confounders might lead to conflicting results [12]. Therefore, we believe that to best assess the effect of lockdown and reduce potential biases, comparing the periods immediately preceding and immediately following lockdown to the lockdown period itself, as we did here, is more reliable than conducting an interrupted time-series analysis comparing the lockdown period with the same period in previous years, even if seasonal effects may introduce bias to this methodology.

We chose to select these six tertiary centers because each of them is the only referral center within their own network of maternity hospitals. This method made it easier to observe any changes in the incidence of preterm births, low birthweights, or stillbirths because these six centers receive referred high-risk women for imminent preterm births and women with fetal growth restrictions, especially before 32 and 34 weeks of gestation. This methodology makes us confident in our results, especially because the results were similar in each of the six tertiary centers. Finally, as highlighted by Goldenberg and McClure, investigators who do not see a reduction in preterm birth are less likely to publish their results [1]. 

### 4.3. Strengths and Limitations

The main strength of this study was its data gathering was broadly geographically distributed across the country. This accounted for the heterogeneity of COVID-19 prevalence in the population. Data were collected prospectively in each maternity hospital, so that missing data accounted for less than 5% of all maternal and perinatal outcomes and provided a significant overview of the impact of the lockdown on pregnancies in France. Furthermore, for all women included in the study, gestational age at delivery was reliable because its determination was based on the results of the first trimester ultrasound scan routinely performed in France, or from oocyte retrieval in ART-generated pregnancies. We were able to report the proportion of spontaneous preterm births (usually defined as those occurring after spontaneous onset of labor or membrane rupture) and of medically indicated preterm births, as well as to classify preterm births rates by gestational age. However, our study had some limitations. We were not able to provide information on the prevalence of women affected by COVID-19 in our population [13]. Indeed, at this period, testing was challenging because accurate screening tests were lacking. Patients in France were not screened for SARS-CoV-2 at admission to delivery rooms during this period. However, the number of pregnant women requiring hospitalization for COVID-19 represented a minority of the population studied in our series, as was the case in France [14]. As our study was also limited by its retrospective design, we cannot exclude the possibility of an unmeasured or unobservable confounding bias. Finally, even if the inclusion of six centers is indeed a strength of this study, it is limited by a relatively low number of deliveries in each center, as well as the short periods which were studied.

## 5. Conclusions

Our study findings do not support the idea that lockdown during the COVID-19 pandemic might decrease the rates of preterm births and low birthweights or increase the rate of stillbirths.

## Figures and Tables

**Table 1:** Comparison of maternal and pregnancy characteristics between the three periods.

	Pre-Lockdown *n* = 4093	Lockdown *n* = 3829	Post-Lockdown *n* = 4007	*p1*	*p2*
Maternal age, years	31.8 (28.0–35.4)	31.8 (28.1–35.5)	31.8 (28.1–35.5)	0.28	0.35
Nulliparous	1960 (49.7%)	1877/3691 (50.8%)	1947/3849 (50.6%)	0.31	0.83
Multiple pregnancies:	133/4093 (3.2%)	115/3829 (3.0%)	132/4007 (3.3%)	0.57	0.50
twins	129/4093 (3.1%)	113/3829 (2.9%)	127/4007 (3.2%)		
Triplets or higher order	5/4093 (0.1%)	3/3829 (0.1%)	6/4007 (0.1%)		
BMI (kg/m^2^)				0.82	0.42
<18	103/3664 (2.8%)	99/3509 (2.8%)	108/3635 (3.0%)		
18–24.9	2227/3664 (59.8%)	2062/3509 (58.7%)	2199/3635 (60.5%)		
25–30	855/3664 (22.9%)	832/3509 (23.7%)	814/3635 (22.4%)		
≥30	536/3664 (14.4%)	516 /3509 (14.7%)	514/3635 (14.1%)		
Active smoking	368/3241 (11.3%)	310/3043 (10.2%)	322/3195 (10.1%)	0.23	0.91

Results are presented as the number and (percentage) or median and interquartile range (Q1–Q3). *p1*: Comparison between lockdown and pre-lockdown periods. *p2*: Comparison between lockdown and post-lockdown periods. BMI, body mass index.

**Table 2:** Comparison of perinatal outcomes between the three periods.

	Pre-Lockdown (*n* = 4093)	Lockdown (*n* = 3829)	Post-Lockdown (*n* = 4007)	*p1*	*p2*
Diabetes	364/4093 (8.9%)	288/3829 (7.7%)	365/4007 (8.8%)	0.17	0.09
Gestational diabetes with insulin	132/4093 (3.2%)	99/3829 (2.8%)	137/4007 (3.4%)		
Preexisting diabetes with insulin	232/4093 (5.7%)	189/3829 (4.9%)	228/4007 (5.6%)		
Hypertensive disorders	220/4093 (5.4%)	216/3829 (5.6%)	204/4006 (5.1%)	0.71	0.30
Gestational age in weeks and days (in superscript).	39.3 (38.2–40.3)	39.3 (38–40)	39.3 (38.2–40.3)		
<28	32/4169 (0.8%)	35/3905 (0.9%)	60/4105 (1.4%)	0.68	0.27
28–31^+6^	70/4169 (1.7%)	87/3905 (2.2%)	72/4105 (1.8%)		
32–36^+6^	184/4169 (4.4%)	173/3905 (4.4%)	176/4105 (4.3%)		
37–40^+6^	3506/4169 (84.1%)	3270/3905 (83.7%)	3430/4105 (83.6%)		
≥41 weeks	377/4169 (9.0%)	340/3905 (8.7%)	367/4105 (8.9%)		
Birthweight					
VLBW < 1500 g	102/3833 (2.6%)	127/3578 (3.5%)	135/3784 (3.6%)	0.03	1
LBW < 2500 g	460/3833 (12.0%)	464/3578 (12.8%)	470/3784 (12.4%)	0.22	0.50
Stillbirth	22/4093 (0.5%)	20/3829 (0.5%)	15/4007 (0.4%)	0.81	0.41

VLBW, very low birthweight; LBW, low birthweight. Results are presented as the number and (percentage). *p1*: Comparison between lockdown and pre-lockdown periods. *p2*: Comparison between lockdown and post-lockdown periods.

**Table 3:** Adjusted odds ratios (aORs) and 95% confidence intervals (CIs) for perinatal outcomes.

	Pre- vs. Lockdown Period		Post- vs. Lockdown Period	
	aOR (Ref = pre) and 95% CI	*p*	aOR (Ref = after) and 95% CI	*p*
Gestational Age				
<32 weeks of gestation	0.90 (0.69–1.19)	0.47	1.04 (0.80–1.36)	0.74
<37 weeks of gestation	1.00 (0.86–1.16)	0.98	0.95 (0.81–1.10)	0.47
Birthweight				
VLBW < 1500 g	0.84 (0.64–1.10)	0.22	0.97 (0.74–1.27)	0.85
LBW < 2500 g	0.97 (0.84-1.13)	0.72	0.94 (0.81–1.10)	0.47
Stillbirths *	0.81 (0.39–1.37)	0.34	1.05 (0.52–2.12)	0.52

VLBW, very low birthweight; LBW, low birthweight. * Adjusted for place of birth, maternal age, multiparity, multiple pregnancies, diabetes, and hypertensive disorders.

**Table 4:** Elective versus spontaneous deliveries within each group for preterm birth and low birthweight infants.

	Pre-Lockdown	Lockdown	Post-Lockdown	*p1*	*p2*
Preterm birth < 37 weeks				0.05	0.91
Spontaneous delivery	46.3% (199/429)	40.0% (161/402)	40.4% (159/393)		
Elective delivery	53.6% (230/429)	53.6% (230/429)	53.6% (230/429)		
Preterm birth < 32 weeks				0.63	0.52
Spontaneous delivery	50.8% (57/112)	42.6% (52/122)	36.8% (46/125)		
Elective delivery	Elective delivery	Elective delivery	Elective delivery		
Birthweight ≤ 2500 g				0.70	0.71
Spontaneous delivery	36.3% (167/460)	37.5% (174/464)	35.5% (167/470)		
Elective delivery	Elective delivery	Elective delivery	Elective delivery		
Birthweight ≤ 1500 g				0.17	0.14
Spontaneous delivery	39.2% (40/102)	41.7% (53/127)	31.2% (39/125)		
Elective delivery	Elective delivery	Elective delivery	Elective delivery		

Elective delivery: delivery after induction of labor or pre-labor caesarean delivery. *p1:* Comparison for each outcome between the pre-lockdown period and the lockdown period. *p2:* Comparison for each outcome between the post-lockdown period and the lockdown period.

## Data Availability

Data are available on request due to restrictions, e.g., privacy or ethical.
